# Gastroesophageal reflux disease in a typical African population: a symptom-based multicenter study

**DOI:** 10.1186/s12876-020-01261-8

**Published:** 2020-04-15

**Authors:** Sylvester Chuks Nwokediuko, Olusegun Adekanle, Adegboyega Akere, Abdulfatai Olokoba, Chiedozie Anyanechi, Sabo Mustapha Umar, Abubakar Maiyaki, Uchenna Ijoma, Olive Obienu, Augustine Uhunmwangho, Dennis Ndububa

**Affiliations:** 1 Department of Medicine, University of Nigeria Teaching Hospital, Ituku Ozalla, Enugu State Nigeria; 2 Department of Medicine, Obafemi Awolowo University Teaching Hospital Complex, Ile-Ife, Osun State Nigeria; 3 Department of Medicine, University College Hospital, Ibadan, Oyo State Nigeria; 4 Department of Medicine, University of Ilorin Teaching Hospital, Ilorin, Kwara State Nigeria; 5 Department of Medicine, Federal Medical Centre, Umuahia, Abia State Nigeria; 6 Department of Medicine, Abubakar Tafawa Balewa University Teaching Hospital, Bauchi, Bauchi State Nigeria; 7 Department of Medicine, Othman Dan Fodio University Teaching Hospital, Sokoto, Sokoto State Nigeria; 8 Department of Medicine, University of Abuja Teaching Hospital, Gwagwalada, Nigeria

**Keywords:** GERD, Nigeria, Africa

## Abstract

**Background:**

The prevalence of gastroesophageal reflux disease (GERD) in Africa is not known but is believed to be increasing because of demographic and epidemiologic transition. The main objectives of this study were to determine the prevalence and risk factors of GERD, and its degree of overlap with dyspepsia and irritable bowel syndrome (IBS) in Nigeria, a typical African population.

**Methods:**

This was an observational, cross-sectional and descriptive study of adult Nigerians. Diagnosis of GERD was by means of the gastroesophageal reflux disease questionnaire (GERDQ) while the diagnosis of dyspepsia and IBS was based on the Rome III criteria for the diagnosis of functional gastrointestinal disorders. The GERDQ and Rome III questionnaires for dyspepsia and IBS were merged into a composite questionnaire and administered to the study participants who were recruited with a multi-stage sampling technique.

**Results:**

Out of 3520 subjects who participated in the study across the country, 269 (7.6%) satisfied the diagnostic criteria for GERD, while 107 (3.0%) had GERD associated with significant impairment of quality of life. Risk factors of GERD (represented by odds ratios) were age 1.014(95% CI: 1.006–1.022), use of analgesics 1.461 (95% CI: 1.060–2.025), and use of herbs 1.318 (95% CI: 1.020–1.704). Overlap of GERD with dyspepsia and/or IBS was observed in over 50% of cases.

**Conclusions:**

The prevalence of GERD in this study is 7.6%. Age, use of analgesics and use of herbs increase the risk, albeit minimally. A high degree of overlap with dyspepsia and IBS exists in Nigerian patients with GERD.

## Background

The definition of gastroesophageal reflux disease (GERD) remains work in progress. However, the Montreal consensus definition is the most widely used, being symptom-based and patient-centered. It defines GERD as symptoms and/or complications resulting from the reflux of gastric contents into the esophagus, up to the mouth, and possibly lungs [1]. The two cardinal symptoms of GERD are heartburn and regurgitation, but their sensitivity in diagnosing GERD is suboptimal [2, 3]. Over the years the management of GERD has been underpinned by acid suppression, which has not met the needs of patients. Current knowledge suggests that the mechanisms for generation of these symptoms are heterogeneous [4].

Despite these shortcomings, symptom-based approach remains a pragmatic way of defining and diagnosing GERD and is endorsed by societal guidelines [5, 6]. It has also proven to be useful as initial diagnostic approach for primary care as well as being an effective healthcare cost-saving strategy.

The global burden of GERD is huge but prevalence depends on the diagnostic criteria used. In a landmark meta-analysis by El-Serag et al. in 2014, the disease affected 18 to 28% of the population in North America, 9 to 26% in Europe and 3 to 8% in East Asia [7]. In a more recent study on GERD prevalence and risk factors on a global scale, the prevalence varied according to country from 2.5% in a Chinese to 51.2% in a Greek study [8]. Whereas the validated gastroesophageal reflux disease questionnaire (GERDQ) was used in the Chinese study, the Greek study employed the reflux symptom index [9, 10]. In the Persian gulf and Middle East regions, higher rates have been reported; 18 to 21% in Iran, 19 to 25% in Turkey and 29% in Saudi Arabia [11–14].

The prevalence of GERD in Africa is not known, and may actually be on the increase along with the trend in western world and Asia because of the obesity epidemic, advancing age, changes in diet and sedentary lifestyle. The continent of Africa is conspicuously missing in several attempts at mapping the global epidemiology of GERD [7, 8, 15].

Nigeria is a large West African country located on the gulf of Guinea with a population of about 200 million and diverse culture, making it ideal for a survey intended to reflect the situation in Africa. The country also has a high prevalence of Helicobacter pylori infection, similar to what obtains in other African countries where the isolates are both CagA and VacA positive in over 90% of cases [16].

The main objectives of this study were to determine the prevalence and risk factors associated with GERD in Nigerians, to determine the symptom profile of Nigerians with GERD, and to determine the degree of overlap between GERD symptoms and those of dyspepsia and IBS.

## Methods

The study was observational, cross-sectional and descriptive. A team of 11 gastroenterologists with a wide national spread was raised to carry out the study. Ethical approval was obtained from the research ethics Committee of University of Nigeria Teaching Hospital Ituku/Ozalla. The study area was the Federal Republic of Nigeria and adults of 18 years and above constituted the study population.

For the purpose of sampling, a multi-stage sampling technique was employed. The country was divided into North and South. By simple random sampling, two geopolitical zones from the North and 2 from the South were used for further sampling. From each geopolitical zone, 2 states were picked by simple random sampling, and from each state 2 Local Government Areas (LGA) were picked and from each LGA, 2 communities or clusters were picked, all by simple random sampling. A pilot survey was carried out in 2 communities during which the study questionnaire was validated. Advocacy visits were arranged to traditional rulers, market leaders and religious leaders who eventually facilitated data collection. Resident doctors from the institutions of affiliation of the investigators were trained to serve as research assistants and they assisted in data collection. Data collection lasted from June 1, 2017 to December 31, 2017.

The GERDQ and the dyspepsia and IBS modules of Rome III diagnostic criteria for the diagnosis of functional gastrointestinal disorders were merged into a composite questionnaire which was used for the study [17, 18]. According to Rome III, dyspepsia is the presence of one or more symptoms considered to originate from the gastroduodenal region. These include bothersome postprandial fullness, early satiation, epigastric pain and epigastric burning for at least 3 months, with onset at least 6 months previously. Bothersome postprandial fullness and early satiation make up postprandial distress syndrome (PDS), while epigastric pain and epigastric burning constitute epigastric pain syndrome (EPS). IBS is recurrent abdominal pain or discomfort at least 3 days per month for the past 3 months, with symptom onset greater than 6 months before diagnosis, associated with 2 or more of the following:

- Improvement with defecation
- Change in frequency of stool
- Change in stool form (appearance).

The GERDQ is a 6-item questionnaire for the diagnosis and management of GERD. The questions relate to the experience of the respondent in the last 7 days. The first 4 questions deal with diagnosis while the last 2 determine the impact of GERD on quality of life. Each response attracts a numerical score and the scores are summed at the end. Scores below 8 suggest low probability of GERD, while scores ≥8 suggest GERD.

The Dyspepsia module of Rome III diagnostic questionnaire is an 18-item questionnaire with scores assigned to the possible responses to the questions. The scores recorded for specific questions in the module determine whether a respondent would be classified as no dyspepsia, epigastric pain syndrome (EPS), postprandial distress syndrome (PDS), or a combination of EPS and PDS. The IBS module is a 10-item questionnaire in which the score obtained for specific questions determines whether the subject would be classified as no IBS or any of the 4 subtypes of IBS.

Also included in the questionnaire were GERD putative risk factors, including age, gender, occupation, rural/urban residence, religion, tribe, weight, height, body mass index (BMI), waist:hip ratio and dinner time. Alcohol consumption, use of kolanut, coffee, non steroidal anti-inflammatory drugs/analgesics and medicinal herbs were also included, but graded as one of 4 possible responses (Nil, once a month, once a week, and > once a week). On an appointed day the research team came to the research site (markets, village squares, and places of worship). All participants who showed up and consented to participate were included. Physical measurements were also performed on the participants including height, weight, waist circumference, hip circumference, BMI and waist:hip ratio. Pregnant women were excluded from participating in the study.

Statistics was done using Number Cruncher Statistical Software (NCSS) version 10 (NCSS, LLC,USA, www.ncss.com) and GraphPad Prism version 6 (GraphPad Software Inc. USA, www.graphpad.com). Quantitative variables were described as means ± standard deviation (SD), while categorical variables were described as proportions. The association between GERD and putative risk factors was evaluated using univariate and multivariate regression models to derive odds ratios (OR) with 95% confidence interval.

## Results

Out of the 3520 subjects (45.1% males, 68.7% urban dwellers) who participated in the study, 269 (7.6%) had GERD while 107 (3.0%) had GERD with impairment of quality of life (Table 1). The prevalence in the geopolitical zones were: North Central 6.2%, North East 9.8%, South East 7.9, and South West 6.1%. The prevalence in Northern Nigeria (North Central and North East) was 8.6% while the prevalence in Southern Nigeria (South East and South West) was 6.9%. The difference between the prevalence in the Northern and Southern parts of the country was not statistically significant (Fischer’s Exact test, *P* = 0.08). Table 2 shows the anthropometric measurements in all the study participants. The median age of subjects with GERD was 48 years while the median age of those without GERD was 39 years (Table 3). The difference between the 2 medians was statistically significant (Mann Whitney, *P*<0.0001).

**Table 1:** Prevalence of GERD in Nigeria

Region	Prevalence (%)	Count	Sample size
North Central (NC)	6.2	31	502
North East (NE)	9.8	98	999
South East (SE)	7.9	74	934
South West (SW)	6.1	66	1085
Northern Nigeria (NC + NE)	8.6	129	1501
Southern Nigeria (SE + SW)	6.9	140	2019
Nationwide	7.6	269	3520

**Table 2:** Descriptive Statistics (Numerical Data) for all participants

Variable	Mean	Standard Deviation	Median	Interquatile Range
Age (years)	41.9	15.9	40.0	25.0
Weight (kg)	65.4	14.1	65.0	19.0
Height (M)	1.6	0.1	1.6	0.1
Body Mass Index	25.1	9.3	24.2	6.0
Waist (CM)	80.9	19.0	83.0	18.0
Hip (CM)	90.9	20.2	94.0	17.0
Waist/Hip ratio	1.1	4.0	0.9	0.1

**Table 3:** Comparison between GERD and Non-GERD subjects

Variable	GERD	Non-GERD	Mann Whitney	***P*** value
**Age**			194,825	<0.0001*
Mean (SD)	47.3 (16.0)	41.32 (15.8)		
Median (IQR)	48 (26)	39 (24		
**Weight**			255,607	0.5797
Mean (SD)	65.9 (15.5)	65.4 (13.9)		
Median (IQR)	65 (23)	65 (18)		
**Height**			219,445	0.0012*
Mean (SD)	1.7 (13.9)	2.3 (10)		
Median (IQR)	1.6 (0.1)	1.6 (0.1)		
**BMI**			246,841	0.5601
Mean (SD)	25.3 (6.5)	25.1 (9.5)		
Median (IQR)	24.5 (7.3)	24.2 (5.9)		
**W:H**			223,329	0.0387*
Mean (SD)	1.0 (0.8)	1.0 (3.2)		
Median (IQR)	0.9 (0.1)	0.9 (0.1)		

*SD* Standard Deviation, *IQR* Interquartile Range, *W:H* Waist to Hip Ratio

* Statistically significant (Mann Whitney)

Heartburn and regurgitation were recorded in 75.3 and 67.3% respectively. The frequency of heartburn and regurgitation did not differ significantly across the geopolitical zones (Fischer’s Exact test: *P* = 0.2146). Atypical and extra-esophageal symptoms in the study population were chest pain 34.2%, cough 26.0%, voice changes 17.3%, frequent clearing of throat 21.8%, asthma 5.4%, and dental problems 13.0% (Table 4).

**Table 4:** Frequency of typical and atypical symptoms of GERD across geo-political zones in Nigeria

Symptom			Prevalence (%)		
	South East	South West	North Central	North East	Overall
Heartburn	87.8	59.1	80.7	73.4	75.3
Regurgitation	67.6	62.1	77.4	62.2	67.3
Chest pain	54.0	37.9	41.9	3.1	34.2
Cough	33.8	34.9	32.3	3.1	26.0
Voice change	29.7	22.7	16.1	2.0	17.4
Frequent throat clearing	25.7	30.3	29.0	2.0	21.8
Asthma	14.9	4.6	0.0	2.0	5.4
Dental problem	21.6	30.3	0.0	0.0	13.0

On multivariate analysis, the strongest risk factors of GERD, shown by their odds ratios were age 1.014(95% CI: 1.006–1.022), use of analgesics 1.461 (95% CI: 1.060–2.025), and use of herbs 1.318 (95% CI: 1.020–1.704) – (Table 5).

**Table 5:** Logistic Regression of Risk Factors for GERD

Variable	Univariate Logistic Regression				Multivariate Logistic Regression			
		95% CI				95% CI		
	OR	Lower	Upper	*P* value	OR	Lower	Upper	*P* value
Age	1.018	1.011	1.025*	0.000	1.014	1.006	1.022	001
Bmi	1.002	.992	1.013	0.651	1.003	.993	1.013	.574
Cigarette	1.226	.759	1.980	0.405	1.512	.884	2.558	.123
Alcohol	0.817	.608	1.099	0.182	.853	.611	1.191	.351
Cola	1.513	1.194	1.915*	0.001	1.290	.988	1.685	.062
Coffee	1.201	.923	1.562	0.173	1.042	.760	1.428	.798
Analgesics	1.822	1.347	2.466*	0.000	1.461	1.060	2.025*	.021
Herbs	1.502	1.188	1.898*	0.001	1.318	1.020	1.704*	.035
Occupation								
Student	0.771	.482	1.235	0.280	1.101	.417	2.907	.846
Farming	2.236	1.609	3.106*	0.000	1.858	0.743	4.647	.186
Trading/business	0.638	0.498	0.818*	0.000	.745	.309	1.797	.512
Artisan	0.524	0.339	0.811*	0.004	.692	.267	1.795	.449
Civil servant	.988	.717	1.363	0.943	0.991	.402	2.441	.984
Unemployed	3.099	1.606	5.979*	0.001	2.904	0.991	8.507	.052
Others	1.690	1.270	2.249*	0.000	1.538	.633	3.736	.342

* = statistically significant

**Fig. 1:**
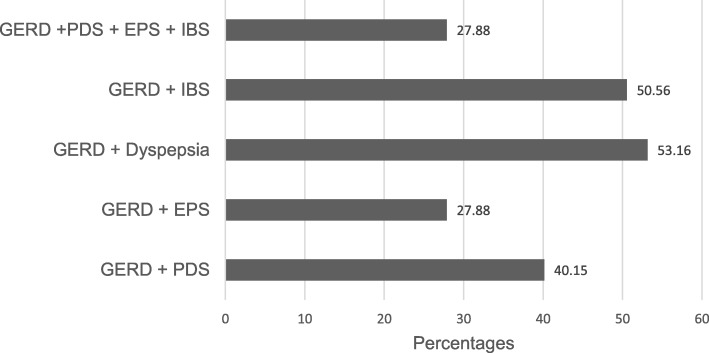
Overlap of GERD with dyspepsia and Irritable bowel syndrome. GERD: Gastroesophageal reflux disease. PDS: Postprandial distress syndrome. EPS: Epigastric pain syndrome. IBS: Irritable bowel syndrome

A high degree of overlap of GERD with dyspepsia (or its subtypes) and IBS was observed in the GERD subjects (Table 6). Overlap was observed with dyspepsia (53.2%), PDS in 40.2%, EPS in 27.9%, IBS in 50.6% and dyspepsia + IBS in 27.9%. Fig. 1 illustrates the frequency of overlap among GERD, dyspepsia and IBS.

**Table 6:** Overlaps of GERD with Dyspepsia and IBS

Disease Status	Number of cases (%) per region			
(Overlap)	NC	NE	SE	SW
GERD + PDS	7 (22.6)	72 (73.5)	25 (33.8)	4 (6.1)
GERD + EPS	10 (32.3)	23 (23.5)	36 (48.7)	6 (9.1)
GERD+ Dyspepsia	13 (41.9)	79 (80.6)	42 (56.8)	9 (13.6)
GERD + IBS	15 (48.4)	87 (88.8)	29 (39.2)	5 (7.6)
GERD+PDS + EPS+ IBS	4 (12.9)	17 (17.4)	44 (59.5)	10 (15.2)
No of GERD Cases	31	98	74	66
Sample Size	502	999	934	1085

*GERD* Gastroesophageal reflux disease, *PDS* Postprandial distress syndrome, *EPS* Epigastric pain syndrome, *IBS* Irritable bowel syndrome, *NC* North Central, *NE* North East, *SE* South East, *SW* South West

## Discussion

The overall prevalence of GERD in this study was 7.6%. When compared to the global pattern, it appears that this prevalence is closer to what obtains in Asia (2.5 to 7.8%) but farther from the situation in America (18.1 to 27.8%) and Europe (8.8 to 25.9%) [7, 8]. Two possible explanations for the modest GERD prevalence in this study are the roles of Helicobacter pylori infection and obesity.

Helicobacter pylori infection exhibits a striking regional variation across the globe. In a systematic review, sub-Saharan Africa and most of South East Asia have very high prevalence (over 70%) while North America and Western Europe have lower rates of less than 40% [19]. Some epidemiological studies have reported an inverse relationship between Helicobacter pylori infection and GERD [20, 21]. This observation is most marked in populations infected by the cytotoxin-associated gene product (CagA)-positive strains of the organism [22]. Conversely, a group of researchers from Norway reported that Helicobacter pylori infection did not affect the occurrence of reflux symptoms regardless of the cagA status [23]. Furthermore, other studies have shown that eradication of Helicobacter pylori does not cause or exacerbate GERD [24, 25]. This calls for population-based studies in Africa to further elucidate the nature of this relationship.

Obesity is one of the risk factors of GERD and partly accounts for the high prevalence of the latter in North America and Western Europe where it has assumed public health dimensions. Weight gain and high BMI are associated with increased risk of GERD [26]. The relatively lower prevalence of GERD in this study, compared with North America and Western Europe may be related to the much lower prevalence of obesity in Nigeria. The median BMI of all the study participants was 24.2 kg/M^2^ (Table 2) while the median BMI of those with GERD was 24.5 kg/M^2^ (Table 3). It is therefore not surprising that the OR of BMI in the multivariate regression analysis was 1.0 (Table 5). However, with changes in diet to processed and high calorie foods, sedentary lifestyle, inadequate exercise, and mechanized transport, the prevalence of obesity in Africa is actually on the upward trend and GERD is expected follow the same trend [27].

Multivariate regression showed that age, use of analgesics and use of herbs were significant risk factors of GERD though their effects were modest. Over the years, several studies have consistently associated aging with increased risk of GERD [28–32]. A population-based survey of GERD in a region with high prevalence of esophageal cancer showed that age is an independent predictor of non-erosive reflux disease and reflux esophagitis [33]. Severe esophagitis has also been shown to be more common with advancing age. Possible mechanisms for the heightened risk of GERD in the elderly include reduced esophageal motility, impaired salivary and bicarbonate secretion, reduced lower esophageal sphincter pressure, weakness of the diaphragm, high incidence of hiatal hernia and greater likelihood of co-morbidities such as diabetes mellitus and parkinsonism [34]. For reason of these co-morbidities, the elderly is also likely to be on concomitant medications like nitrates, calcium channel blockers and theophylline, which are known to be refluxogenic. Since GERD is a chronic disease, it has been more convenient for researchers to use prevalence as a measure of its morbidity. Prevalence estimates are notorious for being confounded by cohort effect with the result that the observed prevalence may be increased in the elderly. A recent US population-based study reported an increased prevalence of GERD in the aging population and also showed that the greatest increase was observed in the 30–39 year age group [35]. The latter observation was explained by the role of obesity, decreased Helicobacter pylori prevalence, smoking and heavy alcohol consumption. The situation in Africa needs further studies to determine if GERD is becoming more prevalent in the younger population.

The association between analgesics and GERD in this study was modest but may be an important finding. Pain is one of the commonest reasons for seeking medical attention. In the African setting self-medication is rampant. In one questionnaire study, over 70% of respondents used analgesics of various types, particularly NSAIDs without prescription [36]. The use of NSAIDs in Nigeria is characterized by a high degree of self-medication, misuse, use in combination with several other drugs (polypharmacy), prolonged use and use in the elderly [37]. NSAIDs and steroids are the most commonly implicated agents in adverse drug reactions in the Nigerian elderly, with the gastrointestinal tract being the most commonly affected organ [38].

The role of NSAIDs in the causation of GERD has been reported in several studies. In an observational study of French adults, NSAID or aspirin use was a significant risk factor for GERD symptoms [39]. Similar studies from different parts of the globe, including US and UK showed that GERD symptoms were more common in NSAID users than in non-users [40–43]. However, a recent study from Moscow reported that NSAID use did not affect the prevalence of GERD [44]. This calls for more studies to unravel the real nature of the relationship.

Another important observation in this study is the association between GERD and use of herbal medicines. Use of herbal medicine is an age-long practice in Africa, and there is currently a growing menace of this practice in Nigeria. A study of urban residents in Lagos showed that herbal medicines were reportedly used by 67% of respondents [45]. Granted that some of the herbs have potentials for phytotherapeutic applications, contaminants like heavy metals and microbes pose dangers of toxicity and infection [46]. Furthermore, alcohol, which is often added to these herbal medicines has been shown to predispose to reflux by direct toxicity to esophageal mucosa and relaxation of the lower esophageal sphincter [47, 48]. Other problems associated with these herbs include inadequate knowledge of their mechanisms of action, possible adverse reactions, contraindications and interactions with other orthodox pharmaceutical products.

A high degree of overlap between GERD, dyspepsia and IBS is another interesting finding in this study (Fig. 1). These disorders of gut-brain interaction, are also called functional gastrointestinal disorders. GERD-dyspepsia overlap was observed in 53.2% of cases, GERD-IBS overlap in 50.6%, and GERD-dyspepsia-IBS overlap in 27.9%. In a meta-analysis, the prevalence of dyspepsia in GERD was 43.9% (95% CI: 35.1–52.9%) with a pooled odds ratio of 6.94 (95% CI: 4.33–11.1) [49]. The phenotypes of GERD include erosive reflux disease and non-erosive reflux disease. Hypersensitive esophagus and functional heartburn are symptomatically similar to GERD but are classified as functional esophageal disorders according to the Rome IV model [50]. Similarly, dyspepsia can be organic or functional. Most dyspeptic patients actually have functional dyspepsia. Based on symptom profile, functional dyspepsia can also be classified into EPS and PDS. Some pathophysiologic mechanisms that have been implicated in GERD also play some role in functional dyspepsia, including visceral hypersensitivity, impaired fundal accommodation, delayed gastric emptying and altered gastrointestinal motility [51, 52]. Psychological factors have also been considered to play an important role in patients with overlapping functional dyspepsia and heartburn. Somatization, anxiety, depression and insomnia are particularly important in this respect [53]. Functional heartburn exhibits higher overlap with dyspepsia than the non-erosive reflux disease phenotype [54]. Similarly, there is a high degree of overlap between GERD and IBS, but functional heartburn overlaps with IBS more often than GERD [55]. Since endoscopy and functional reflux testing were not done in this study, it was not possible to separate our GERD patients into erosive reflux disease, non-erosive reflux disease hypersensitive esophagus and functional heartburn.

The clinical importance of these overlaps is the fact that they significantly worsen disease severity and health-related quality of life, and must be factored into clinical management if diagnostic and therapeutic failures are to be minimized or eliminated in this category of patients [56]. This is because some treatment guidelines are disease-specific with little or no attention to overlaps. In the same vein these overlapping disorders can act as confounders in clinical trials.

### Limitations of study

The participants in the study were made to come to places of worship, markets and village squares to take part. This means that some people who could not come out from their homes for one reason or another were not captured. Probably, only the very active and healthy participated and that may have affected the result. In future studies, the investigators should visit the communities and households and carry out the study in the places of abode of the participants in order to capture the healthy and not so healthy members of the community. This would reflect the actual population characteristics.

The questionnaires employed in this study are meant for self-administration. However in the Nigerian setting the low literacy rate (which is below 30% in some regions) made self- administration inexpedient. Also the questionnaires were not translated into local languages because of the multiplicity of languages. Nigeria has more than 500 languages but 12 of them are major.

## Conclusion

The prevalence of GERD in this study is 7.6%. This figure lies within the range that exists in Asia but is much lower than the prevalence in North America and Western Europe. Age, use of analgesics and use of herbs were the independent predictors of GERD, though their effects were modest. Overlap of GERD with dyspepsia and/or IBS was observed in over 50% of cases.

## Data Availability

The datasets used and/or analyzed during the current study are available from the corresponding author on reasonable request.

## Abbreviations

BMI`: Body Mass Index
EPS: Epigastric Pain Syndrome
ERD: Erosive Reflux Disease
GERD: Gastroesophageal Reflux Disease
GERDQ: Gastroesophageal Reflux Disease Questionnaire
IBS: Irritable Bowel Syndrome
LGA: Local Government Area
NSAID: Non-steroidal anti-inflammatory drug
OR: Odds Ratio
PDS: Post Prandial Distress Syndrome
SD: Standard Deviation
USA: United States of America
